# Lantibiotic Immunity: Inhibition of Nisin Mediated Pore Formation by NisI

**DOI:** 10.1371/journal.pone.0102246

**Published:** 2014-07-11

**Authors:** Zainab AlKhatib, Marcel Lagedroste, Iris Fey, Diana Kleinschrodt, André Abts, Sander H. J. Smits

**Affiliations:** Institute of Biochemistry, Heinrich Heine University Düsseldorf, Düsseldorf, Germany; Teagasc Food Research Centre, Ireland

## Abstract

Nisin, a 3.4 kDa antimicrobial peptide produced by some *Lactococcus lactis* strains is the most prominent member of the lantibiotic family. Nisin can inhibit cell growth and penetrates the target Gram-positive bacterial membrane by binding to Lipid II, an essential cell wall synthesis precursor. The assembled nisin-Lipid II complex forms pores in the target membrane. To gain immunity against its own-produced nisin, *Lactococcus lactis* is expressing two immunity protein systems, NisI and NisFEG. Here, we show that the NisI expressing strain displays an IC_50_ of 73±10 nM, an 8–10-fold increase when compared to the non-expressing sensitive strain. When the nisin concentration is raised above 70 nM, the cells expressing full-length NisI stop growing rather than being killed. NisI is inhibiting nisin mediated pore formation, even at nisin concentrations up to 1 µM. This effect is induced by the C-terminus of NisI that protects Lipid II. Its deletion showed pore formation again. The expression of NisI in combination with externally added nisin mediates an elongation of the chain length of the *Lactococcus lactis* cocci. While the sensitive strain cell-chains consist mainly of two cells, the NisI expressing cells display a length of up to 20 cells. Both results shed light on the immunity of lantibiotic producer strains, and their survival in high levels of their own lantibiotic in the habitat.

## Introduction

Since the 1920s the heterogeneous group of bacteriocins have become an interesting research topic for different applications *e.g.*, as food preservatives or as antibiotic alternatives [1]. Bacteriocins are small, heat stable ribosomally synthesized peptides showing antimicrobial activity [2], [3]. They are mostly produced by Gram-positive bacteria and mainly act against other Gram–positive species. Therefore, they are candidates with high potential for the treatment of human bacterial infections with multiple resistances against antibiotics like the pathogenic VRE or MRSA strains [4], [5].

Within the group of bacteriocins, there is a large family called lantibiotics [6]. They contain characteristic thioether bridges, called lanthionine rings, which are post-translationally introduced. These lanthionine rings provide a high level of protection against peptide-digesting enzymes, and more importantly ensure high antimicrobial activity against mainly Gram–positive bacteria, which is reflected by the low nanomolar concentration needed to fulfill their activity [7], [8].

Lantibiotics are produced and secreted in a non-active form and are later activated by cleavage of the specific N-terminal leader peptide. These active lantibiotics are able to lyse mainly Gram-positive bacteria and some Gram-negative bacteria strains are also affected [7], [9].

Within the lantibiotic producer strains, the structural genes for biosynthesis, modification, transport across the cellular membrane, as well as regulation are all localized on a single gene cluster [7], [10], [11]. Additionally, genes encoding a lantibiotic specific immunity system are present, preventing the lantibiotics to harm their own producer strain. Although, lantibiotics are grouped in different classes based on their sizes and activities [11], [12], the lantibiotic specific immune system seems to be conserved in all groups [10]. Two functional proteins LanI, a membrane associated protein, and LanFEG, an ABC transporter localized in the cellular membrane [13], are mediating this immunity.

Nisin is the best-known and most extensively studied lantibiotic and different variants are produced by some *Lactococcus lactis* (*L. lactis*) strains [14]. Due to its high bactericidal activity in combination with low toxicity in humans, nisin is used since decades as a natural preservative in the food industry [8]. Active nisin consists of 34 amino acids and contains five lanthionine rings where the first three rings are separated from the last two intertwined rings by a flexible hinge region [15]. The first two rings are able to bind Lipid II and thereby inhibit cell wall synthesis [16], whereas the hinge region and the last two rings, which are intertwined together, are able to flip into the membrane and create pores [9], [17], [18].

The mode of action of nisin has been thoroughly studied since its discovery. It was suggested that nisin kills bacteria by inhibiting cell-wall synthesis *via* binding to Lipid II, as observed for many other lantibiotics. However, the immediate release of small cytoplasmic compounds such as amino acids, ATP or pre-accumulated rubidium from cells [19], highlighted that nisin acts by the distinct permeabilization of the plasma membrane. The nisin–membrane interaction was thus extensively studied, with a focus on the interaction between the cationic nisin peptide and the abundant anionic lipids of the plasma membrane of Gram-positive bacteria [19]–[21]. More recently, it has been shown that nisin uses Lipid II as a ‘docking molecule’ to form pores in a targeted manner with a high efficiency [22], [23]. Here, in presence of Lipid II, the activity of nisin in model membrane systems is increased by three orders in magnitude compared to the activity of nisin against susceptible bacteria. Therefore, nisin can permeabilize membranes by two different mechanisms: I) through a low-affinity permeation mechanism that is only observed in model systems; II) by a much higher nisin-Lipid II-dependent targeted pore-formation mechanism. In the first mode, which requires micromolar concentrations of nisin and presence of anionic lipids in the target membrane, nisin binds to the anionic lipids, and is subsequently inserted in the membrane at the position of the phospholipid head groups [24]–[27]. The accumulation of nisin in the outer lipid leaflet of the target membrane drives the aggregation of nisin monomers, which is followed by the formation of short-lived pore-like structures.

The second mode of action of nisin is dependent on the presence of Lipid II in the membrane (as present in Gram-positive bacteria), and can be described as follows: specific recognition and binding of Lipid II occurs, which is followed by pore assembly and formation. Interestingly, the pores formed by nisin in the presence of Lipid II are much more stable than pores formed in the absence of the lipid [28], [29]. Nisin has a specific transmembrane orientation in the presence of Lipid II, indicating that the formed pores are stable [17]. This also indicates that the role of Lipid II in the nisin-mediated pore-formation is not only just binding. It has been further shown that Lipid II is a constituent of the formed pore, which consists of four Lipid II molecules and eight nisin molecules [18].

In 2006, Hasper *et al.*, proposed an additional, third mode of action of nisin. Here, nisin binds to the pyrophosphate moiety of Lipid II, which is displaced afterwards from its location in Gram-positive bacteria. Since Lipid II is essential for cell wall synthesis, it is therefore localized in the septum, and the binding leads to growth inhibition. This sequestering effect is a distinct mode of bactericidal activity [30]. Nisin also inhibits the outgrowth of bacterial spores, by for example *Bacillus anthracis* and it has been shown that nisin utilizes Lipid II here as target molecule for this inhibition. Furthermore, nisin-mediated membrane disruption is essential to inhibit spore development [31].

The nisin producer *L. lactis* strains are protecting themselves from this high activity of nisin by expressing two protein systems; the lipoprotein NisI and the ABC transporter NisFEG. When both the proteins are expressed, a high level of immunity against nisin, up to ∼750 nM nisin (1000 IU/ml), is provided [32]. Interestingly, both immunity proteins act cooperatively and each of them displays only 10–30% of the full immunity levels when expressed alone [33], [34].

NisI is a 245 amino acids lipoprotein, with a N-terminal signal sequence, which is removed during posttranslational modification, resulting in the anchoring of NisI to the extracellular side of the cytoplasmic membrane [35]. Koponen *et al.* showed that a significant percentage of expressed NisI is secreted which is not anchored in the membrane, thereby is released into the extracellular media [36]. The presence of this “lipid-free” NisI may have a biological function via the binding of nisin molecules before they can interact with the cell surface, therefore acting as an additional mechanism of self-protection [36].

The importance of NisI for the nisin immunity in *L. lactis* cells was observed *via* deletion of the *nisI* gene. The resulting *nisI* knockout was more sensitive to nisin than the corresponding *nisFEG* knockout [37]. This observation lead to the hypothesis that NisI plays a more effective role than NisFEG in the immunity against nisin, although the differences are small [38].

The exact molecular mechanism of NisI involved in providing immunity is still unknown. Takala and his colleagues showed the functional importance of the C-terminus of NisI, *i.e.*, interacting with nisin [39]. Their study identified that a deletion of 21 amino acids at the C-terminus of NisI, reduced the NisI mediated immunity compared to the level observed with full-length NisI. Interestingly, this C-terminal region of NisI is not involved in co-operation with NisFEG, as the truncated NisI still showed a cooperative effect of nisin resistance when co-expressed with NisFEG [39]. Moreover, the replacement of the 21 C-terminal amino acids of the subtilin-specific immunity protein SpaI with the C-terminal 21 amino acids of NisI (SpaI'-NisI') created a protein, which confers immunity against nisin [39], whereas this has not been observed with the full length SpaI protein. Similar function of the C-terminus was observed for PepI, an immunity protein against the lantibiotic Pep5 where the C-terminal part mainly provides immunity, while the N-terminal part is more important for its membrane localization [40].

Although these different lipoproteins NisI, SpaI and PepI are similar in conferring specific immunity against their cognate lantibiotic, no significant homology in their primary sequence was observed.

In this work, we focus on the individual contribution of the lipoprotein NisI towards immunity of *L. lactis* against nisin. By using a fluorescence-based method we reveal that NisI inhibits pore formation even at concentrations up to 1 µM nisin. Furthermore, the presence of nisin and NisI simultaneously induced a reversible long chain formation of the *L. lactis* cells. Furthermore, both these mechanisms allow the survival of the *L. lactis* cells at high nisin concentrations albeit only for a certain period of time.

## Material and Methods

### Cloning of the shuttle vector pNZ-SV

To allow more efficient DNA-manipulation and cloning, the *L. lactis/E. coli* shuttle vector pNZ-SV was created in the first step by standard genetic manipulations as described by Sambrook *et al.* [41]. The *L. lactis* plasmid pNZnisA-E3 [42] was linearized by PCR using the primer pair pNZE3-BglIIfor (GATGCATCGATAGATCTAGTCTTATAAC) and pNZ-BamHIrev (CTAGATCTATCGATGGATCCCTTAACTTAC). With the primer pair pET24aBglIIfor (CTTGCGGTATTCGAGATCTTGCACG) and pET24BamHIrev (CTAAATACATTCAAATATGGATCCGCTC) and using pET24a as template, the coding region of Kan, which confers resistance to Kanamycin in *E. coli*, and the pBR322 origin were amplified. The PCR-products were hydrolysed with BamHI and BglII and then ligated. In the second step, the *nisA*-gene was replaced by the multiple cloning site (MCS) of pET24a using the In-Fusion HD-Cloning Kit according to manufacturer's protocol (Clontech). The vector pNZ-SV-nisA was linearized by PCR using the primer pair pNZ-SV-for (GCTTTCTTTGAACCAAAATTAG) and pNZ-SV-rev (GGTGAGTGCCTCCTTATAAT). The MCS of pET24a was amplified by PCR using the primers MCS-pET24-Inf-for (AAGGAGGCACTCACCGAATTCGAGCTCCGTCGACAAG) and MCS-pET24-Inf-rev (TGGTTCAAAGAAAGCTGTTAGCAGCCGGATCTCAGTG), where both primers exhibited a 15 bp homology to the vector for the In-Fusion reaction. Restriction analyses and sequencing verified the correct sequence in the resulting plasmid pNZ-SV.

### Cloning of pNZ-SV-nisI and pNZ-SV-nisIΔ22

The *nisI* gene was amplified from the genome of *L. lactis* NZ9700 by PCR and inserted into the pNZ-SV by In-Fusion HD Cloning. The used primers were pNZ-nisI-for (AAGGAGGCACTCACCATGAGAAGATATTTAATACTTATTGTGGCTTAATAG) and pNZ-nisI-rev (TGGTTCAAAGAAAGCCTAGTTTCCTACCTTCGTTGCAAGCTTAAAAT). The ends of the *nisI*-PCR product contained a 15 bp homology overhang to the pnZ-SV vector. After linearization of the vector pNZ-SV by PCR (primers: pNZ-for (GCTTTCTTTGAACCAAAATTAGAAAAC) and pNZ-rev (GGTGAGTGCCTCCTTATAATTTATTT)), the In-Fusion reaction was carried out according to the recommended conditions mentioned by the manufacturer. Site-Directed mutagenesis was used to delete the last 22 amino acids of the C-terminal NisI protein by using two primers: the pNZ*nisIΔ22aa*–for (CCATTCTATTAGAGGAAAATAGCTTACTGAAGCATTTG) and the complement primer as a pNZ *nisIΔ22aa*–rev. The resulting *nisI* variant was called *nisIΔ22* and was verified by sequencing. After the successful cloning of pNZ-SV-*nisI* and pNZ-SV-*nisIΔ22*, the plasmids were transformed into *L. lactis* NZ9000 by electroporation at 1 kV, 25 µF, 5.0 ms, and the corresponding strains were termed NZ9000NisI and NZ9000NisIΔ22. An empty vector pNZ-SV was also transformed into the NZ9000 strain and was used as a control (that excludes any possible effect of the plasmid), and this strain was called NZ9000Erm. Transformation was performed as previously described [43].

### Expression of NisI and NisIΔ22 in *L. lactis* NZ9000

The NZ9000NisI or NZ9000NisIΔ22 strain was grown in GM17 media supplemented with 5 µg/ml erythromycin to an OD_600_ of 0.8. The expression was induced by the addition of nisin (at a final concentration of 1 ng/ml) and the culture was further grown overnight. These cells were then used for the assays described below.

To analyse the expression, the cells were harvested at OD_600_ of 2.0 by centrifuging at 5000×g for 30 min. The resulting pellet was then suspended in 1 ml of buffer containing 50 mM HEPES pH 8.0, 150 mM NaCl, 10% (w/v) glycerol, and 700 kU/ml lysozyme and was incubated 30 min at 37°C followed by 5 minutes at 50°C, allowing lysozyme to lyse the cell wall. Afterwards the buffer with lysozyme was removed by additional centrifugation step and the pellet was resuspended in SDS-loading dye (0.2 M Tris-HCl, pH 6.8, 10% (w/v) SDS, 40% (v/v) glycerol, 0.02% (w/v) bromophenol and β-mercaptoethanol) and analysed *via* SDS-PAGE analysis. Western blot analysis was carried out using a polyclonal antibody against NisI (Eurogentec).

### Purification of nisin

Nisin was purified as described by Abts et al 2011 [44].

Briefly, commercially available nisin powder (Sigma) was dissolved in 50 mM lactic acid pH 3. The nisin solution was then purified using 5 ml HiTrap SP HP cation exchange column (GE Healthcare) pre-equilibrated with the same buffer. Nisin was eluted with 400 mM NaCl and monitored online at a wavelength of 215 nm, since nisin lacks aromatic amino acids in its sequence. In the last step, nisin was precipitated by TCA and dried out after washing it with cold acetone [44]. The concentration of nisin was measured by using RP-HPLC [45].

### Determination the activity of nisin by IC_50_

Cells from the different expressing strains were grown overnight in GM17 media supplemented with 5 µg/ml erythromycin in presence of 1 ng/ml nisin. The diluted cells (final OD_600_ was 0.1) were incubated with a serial dilution of nisin in a 96 well plate. The total volume in each well was 200 µl, consisting of 50 µl nisin and 150 µl GM17 containing the corresponding *L. lactis* strain. The highest concentration of nisin used was adapted to the corresponding maximum immunity displayed by each strain.

The plate was then incubated at 30°C and after 5 hours, the optical density was measured at 620 nm *via* 96-well plate reader BMG. The normalized optical density was plotted against the logarithm of the nisin concentration in order to calculate the IC_50_ of nisin and the data was evaluated using the following equation:
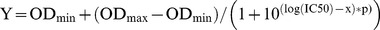
(1)


The OD_max_ value describes the normalized optical density value where no nisin was added, while the OD_min_ value corresponds to the normalized optical density of the cells grown in the highest nisin concentrations. Y represents the resulted normalized optical density value and X represents the logarithmic of the nisin concentration added. The IC_50_ value is thus the concentration of nisin where the growth of the *L. lactis* strain is inhibited by 50% [44].

### SYTOx green nucleic acids binding assay

SYTOx green nucleic acids binding dye possesses a high binding affinity towards nucleic acids. It enters cells only when they contain a pore in the plasma membrane and never cross the intact membranes of living cells [46].

The cells of NZ9000Emr, NZ9000NisI, NZ9000NisIΔ22 were grown overnight in GM17 supplemented with 5 µg/ml erythromycin in presence of 1 ng/ml nisin. The overnight culture was diluted to an OD_600_ of 0.1 in fresh media supplemented with 5 µg/ml erythromycin. The cultures were further grown until the OD_600_ reached 0.5, the SYTOx green dye was then added at a final concentration of 5 µM and incubated for 5 minutes according to the manual provided by the manufacturer (Invitrogen). The fluorescence signal, which was measured at an excitation and emission wavelength of 504 nm and 523 nm, respectively, was monitored for 400 seconds to obtain a stable baseline. At 400 seconds nisin was added and the fluorescence was monitored for further 15 minutes. The SYTOx green experiment was performed in at least triplicates with three differently purified nisin bactches each.

### Regrowth experiment of the different *L. lactis* strains

Cells of NZ9000Erm, NZ9000NisI and NZ9000NisIΔ22aa were grown overnight in GM17 media. A main culture was inoculated using overnight culture to an OD_600_ of 0.1. After a 30 min pre-incubation at 30°C, the GM17 broth was supplemented with 0 (control) or 10-fold IC_50_ concentration (nM) of nisin and the cells were incubated for 1, 2, 3, 4 and 5 h. Cells (out of 3 ml GM17 medium) were harvested and the cell pellet was washed three times with fresh GM17 medium. The recovered cells were used to inoculate fresh GM17 medium to OD_600_ of 0.1 and incubated at 30°C in 96-well plate (vol. 200 µl) for a maximum of 15 hours. To count the number of living cells, 100 µl (after a 1∶100 or 1∶10.000 dilution) of every sample was plated on GM17 agar plates supplemented with 5 µg/ml erythromycin and incubated at 30°C for two days. The grown colonies were subsequently counted.

### Morphology study

To study the effect of nisin on cell morphology, the overnight culture was diluted to an OD_600_ of 0.1. The cells were incubated with 0, 1, 10, 30 nM of nisin for 3 hours and then were harvested at 13.000 rpm for 15 min. Harvested cells were washed with PBS buffer (50 mM phosphate buffer pH 7.2, 150 mM NaCl) and fixed with a 1∶1 mixture of absolute ethanol and PBS buffer. Afterwards 10 µl of the fixed cells were applied to poly-L-lysine cover slides. Followed by the addition of 5 µl of mounting medium, the sample was dried before use. For long-term storage, nail polish was used to seal the cover slips. These samples were monitored using a Nikon Eclipse Ti inverted microscope with a CFI60 100x/1.35 oil objective. The phase contrast pictures were obtained after a raster scan of 8×8 pictures with 5 areas per sample. The Nikon Nis-Elements imaging software was used to control the microscope and the imaging software ImageJ Version 1.47 was used for analysis.

## Results

### Activity of NisI and NisIΔ22 in *L. lactis*

**Figure 1:**
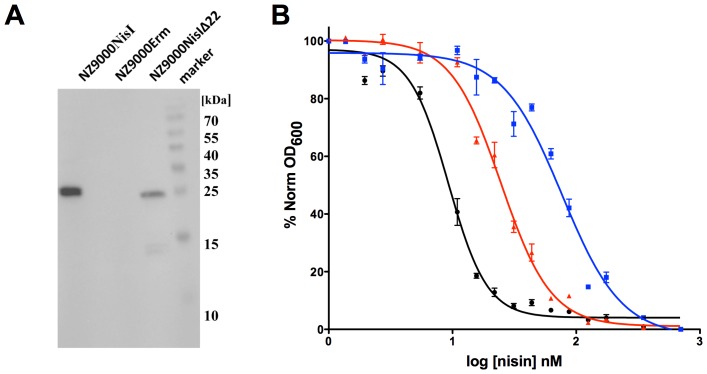
Western blot analysis using a polyclonal NisI antibody (A). Shown are the *L. lactis* strains: NZ9000Erm, NZ9000NisI and NZ9000NisIΔ22 strain. **IC_50_ determination of different strains (B)**. Growth inhibition experiments were performed with nisin using different strains. Black line: NZ9000Erm strain; blue line: NZ9000NisI strain; red line: NZ9000NisIΔ22 strain. Data was fitted and evaluated according to equation (1). Each experiment was performed at least in triplicates.

The *nisI* gene was cloned in a pNZ-SV vector, which was complemented with an origin of replication for *E. coli*, allowing rapid cloning and mutagenesis in all standard *E. coli* laboratory strains. After successful cloning, the plasmids were transformed into *L. lactis* for homologous expression of the NisI or NisIΔ22 protein. *L. lactis* NZ9000 was used, a derivative of the plasmid-cured *L. lactis* MG1363 which contained the *nisRK* genes inserted in the chromosomal pepN locus [47]. This strain is commonly used as the host for the nisin-controlled gene expression system (NICE) [48]. However, since this NZ9000 strain lacks the nisin immunity genes *nisI* and *nisFEG*, it is highly sensitive to nisin [49]. The transformation of an empty plasmid pNZ, a plasmid harbouring wild type NisI and a C-terminal truncation of NisI into *L. lactis* NZ9000 resulted in the strains NZ9000Erm, NZ9000NisI and NZ9000NisIΔ22, respectively. The expression of NisI and NisIΔ22 was monitored by western blot analysis using a polyclonal NisI antibody (see Figure 1A). Here, a slight double band is visible for full-length NisI. The upper band resembles NisI, which is not processed and still containing the secretion signal. It remained inside the cell and therefore does not contribute to the nisin immunity activity of NisI observed below. From this western blot it can be judged that both NisI and NisIΔ22 were expressed in similar quantities.

To quantitatively assess the growth inhibitory activity of nisin, a liquid culture assay was performed using the NZ9000Erm, NZ9000NisI and NZ9000NisIΔ22 strains. The optical density after 5 hours of growth of the corresponding *L. lactis* strain cultures was plotted against the logarithm of the different concentrations of nisin added. Thus, the activity of nisin could be measured and quantified by calculating the amount of nisin required to inhibit cell growth by 50% (IC_50_) using equation 1. In case of the control strain NZ9000Erm, nisin exhibited a high activity (IC_50_ = 9.1±0.7 nM (Figure 1B, black curve)). The NZ9000NisI strain displayed an almost 8–10 fold higher IC_50_ value of 73.0±10.2 nM (Figure 1B, blue curve). The strain expressing NZ9000NisIΔ22 displayed an intermediate IC_50_ value of 25.3±1.7 nM (Figure 1B, red curve). This showed that NisI is capable of conferring immunity and that the C-terminus plays an important role.

### Pore formation by nisin

**Figure 2:**
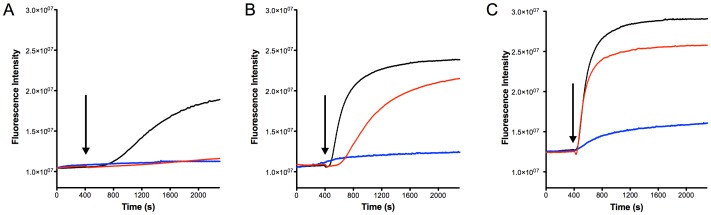
SYTOx green assay to visualize pore formation mediated by nisin. The NZ9000Erm strain (black line), NZ9000NisI strain (blue line) and the NZ9000NisIΔ22 strain (red line) were grown and incubated with the SYTOX green dye. The fluorescence signal was monitored online using a fluorolog (Horiba III). After 400 seconds a stable baseline was reached and nisin was added (A) 10 nM (B) 30 nM and (C) 1000 nM. The addition of nisin is indicated with an arrow. The rapid increase of the fluorescence signal indicated pore formation. The data are representatives of at least three independent measurements.

As mentioned before, nisin has several modes of action. The predominant one being the binding of nisin to Lipid II, a cell wall precursor, which leads to the inhibition of cell growth. Upon binding, nisin is also able to form pores in the membrane, which leads to membrane disruption and subsequently rapid cell death. The latter one can be visualized by a SYTOx green nucleic acid dye. In this assay when pores are formed, the dye enters the cells of *L. lactis* and binds to the DNA resulting in a rapid increase of the fluorescence signal, which can be monitored in real time [46], [50]. To ensure that the *L. lactis* cells were in a good shape, cells were chosen in their exponential growth phase (OD_600_ = 0.5). Different concentrations of nisin were used reflecting concentrations slightly below or above the IC_50_ values of each strain determined above (10, 30 and 1000 nM nisin, Figure 2). The control measurement where no nisin was added (buffer control), no effect on the fluorescence signal could be observed.

Upon incubation of the NZ9000Erm, NZ9000NisI and NZ9000NisIΔ22 strains with the SYTOx green dye, a stable baseline was reached. After 400 seconds, nisin was added (indicated with an arrow in Figure 2) and the fluorescence signal was then monitored continuously.

Upon addition of 10 nM nisin to the NZ9000Erm strain, an increase in the fluorescence was observed starting at 800 seconds and reaching its maximum after 2000 seconds, indicating pore formation in the cytoplasmic membrane (Figure 2A). In contrast, at the same nisin concentrations, no increase in the fluorescence signal was observed for the NZ9000NisI and the NZ9000NisIΔ22 strains, indicating that the SYTOx dye did not enter the cells and thus, no pore formation occurred (Figure 2A). This is in line with the IC_50_ measurement data as a nisin concentration of 10 nM is slightly above the IC_50_ of the NZ9000Erm strain, but below the IC_50_ of the NZ9000NisI and NZ9000NisIΔ22 strains (Table 1).

**Table 1:** IC_50_ values determined for the different strains.

	NZ9000Erm	NZ9000NisI	NZ900NisIΔ22aa
**Nisin**	9.1±0.7 nM	73.0±10.2 nM	25.3±1.7 nM

When 30 nM of nisin was added, the curve of the NZ9000Erm strain increased more rapidly (shortly after addition) and the slope was also steeper (Figure 2B). When compared to the curve obtained upon addition of 10 nM nisin, the maximum of the fluorescence signal was also higher, indicating that more cells were lysed. A similar increase in the fluorescence signal was also observed for the NZ9000NisIΔ22 strain where the signal rapidly increased after roughly 700 seconds. However, at this nisin concentration, the NZ9000NisI strain showed only a minimal increase of 10% of the value observed for the NZ9000Erm strain (Figure 2B). When the nisin concentration was further increased to 1000 nM (which is 100-fold above the IC_50_ for the NZ9000Erm strain 50-fold above the IC_50_ for NZ9000NisIΔ22 strain, and 15-fold above IC_50_ for the NZ9000NisI strain), the curves became even more pronounced. The fluorescence signal observed for the NZ9000Erm strain increased immediately after the addition of nisin (Figure 2C). Similarly, the NZ9000NisIΔ22 strain displayed an increase in the fluorescence signal directly after nisin treatment. However, a different maximum of the fluorescence signal was reached. Interestingly, only a small increase in the fluorescence signal was observed for the NZ9000NisI strain (Figure 2C). The curve shape also indicated towards a gradual effect rather than a sharp and sudden effect, suggesting that this increase cannot be assigned to rapid cell lysis due to a nisin induced pore formation.

From these results, we can conclude that the presence of NisI inhibits the pore formation activity of nisin. This inhibition seems to be a stable effect since even at concentrations 15-fold above the determined IC_50_ values no pore formations could be observed. Furthermore, this inhibition is mediated by the C-terminus, since its deletion displayed pore formation at concentrations higher than the IC_50_ value determined for the NZ9000NisIΔ22 strain.

These results prompted us to re-evaluate our IC_50_ values in more detail. For the NZ9000NisI strain, cell growth was inhibited by 50% when a nisin concentration of 70–80 nM was added. Upon addition of higher concentration of nisin; no growth was observed as measured by the optical density (Figure 1B). Here, it is important to mention that the IC_50_ is measured after 5 hours of growth. As visualized by the SYTOx green assay, no pore formation was observed. Although a slight increase of the fluorescence was observed, suggesting that the NZ9000NisI strain was not suffering from nisin induced pore formation, but rather the strain had stopped growing.

### Recovery experiment: Regrowth of NZ9000NisI

The recovery experiment was performed to determine the ability of *L. lactis* NZ9000NisI cells to re-grow after being exposed to high concentrations of nisin.

**Figure 3:**
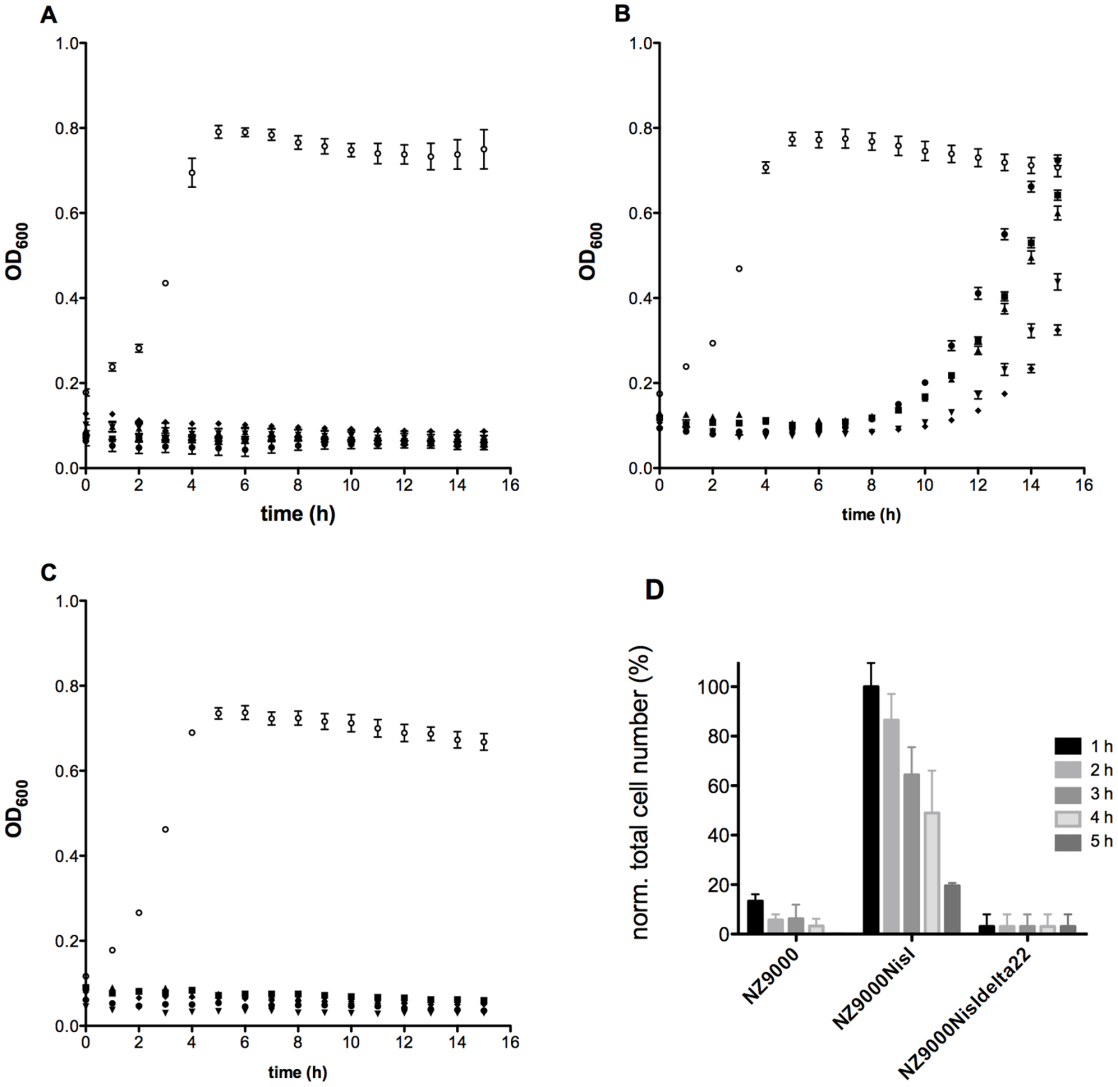
Growth recovery assay. The different strains were incubated for 1 (•), 2 (▪), 3(▴), 4(▾) and 5(⧫) hours at an OD_600_ of 0.1 with nisin at a concentration which represents 10-fold the IC_50_ determined, 100 nM, 300 nM and 600 nM for the NZ9000Erm (A), NZ9000NisIΔ22 (B) and the NZ9000NisI (C) strains, respectively. The cells were separated from the growth media by centrifugation and extensively washed with media to remove the remaining nisin. Afterwards the cells were transferred into fresh medium at a final OD_600_ of 0.1 and the growth was monitored by measuring the optical density at 600 nm. As a control (○) the corresponding strains without the addition of nisin during pre-incubation were used. Each experiment was performed 4 times. Within the different experiments, the interval of recovery comprised between 5 and 8 hours. Furthermore the end point OD_600_ (after 15 hours growth) was in a range of 65–100% recovery ability (compared with the end point OD_600_ of the control). To control the number of cells surviving the incubation with high nisin concentrations, the resuspended cells were striked out on GM17 agar plates. The number of colonies on these plates resemble the total number of living cell in the cell suspension with an OD_600_ of 0.1. A normalisation of the total cell number between the strains NZ9000Erm, NZ9000NisI and NZ9000NisIΔ22 shows the relative distribution depending on the living cells (D). The NZ9000 NisI after 1 h incubation time is set as 100% (total cell number: 261.866±32.809) and reflects the 1.4% of surviving cells compared to the control (total cell number: 24.800.000±1.844.776). Longer incubation times lead to survival rates of 20% for NZ9000NisI. Even fewer cells, only 0.1%, survived for the NZ9000Erm and NZ9000NisIΔ22 strains, when compared to the control. The error bars indicating the standard deviation of three independent experiments.

The NZ9000Erm, NZ9000NisI and NZ9000NisIΔ22 strains were incubated with a nisin concentration 10-fold higher than their corresponding IC_50_ values; 100 nM, 600 nM and 300 nM for the NZ9000Erm, NZ9000NisI and NZ9000NisIΔ22, respectively. As a control, the same strains were used without adding nisin. After incubation of 1, 2, 3, 4 and 5 hours, the cells were harvested by a centrifugation step, extensively washed and then re-suspended to an final OD600 of 0.1 in fresh media lacking nisin. The growth was monitored for further 15 hours by measuring the OD_600_ every hour (Figure 3).

The number of cells re-growing after reducing the nisin concentration was used as a parameter to determine whether the strains were protected against nisin or not.

For both NZ9000Erm and NZ9000NisIΔ22 strains, no growth was observed, indicating that they were killed by nisin (Figure 3A and 3C), while the control of these strains displayed an exponential growth. In contrast, the NZ9000NisI strain started exponential growth, although after a delay time of 5–8 hours (Figure 3B).

This shows that some NZ9000NisI cells could survive in an environment containing high nisin concentrations and could start to regrow again, when transferred into fresh GM17 medium. Furthermore, this effect was dependent on the incubation time. When NZ9000NisI cells were incubated for 1–3 hours they started growing after 3 hours whereas the potential of the cells to regrow was reduced, when the cells were incubated for longer times (4–5 hours). This was also observed when looking at the final OD_600_ of the cells, resulting in values of 0.7, 0.64 and 0.6, whens cells were incubated for 1, 2 and 3 hours, respectively with high nisin concentrations. The cells incubated for longer time (4 and 5 h incubation with nisin) did not grow and showed a final OD_600_ of 0.4 and 0.3, (Figure 3B). The cells incubated for only a shorter time period, reached a similar OD_600_ as the cells without incubation with nisin, indicating that they were fully recovered. The long lag-phase in the growth curve indicated that not all NZ9000NisI cells survived the treatment, with a nisin concentration corresponding to 10-fold IC_50_ value, suggesting that the OD_600_ of 0.1 included not only just living cells but also dead cells. Therefore, we plated a fraction of the cells onto agar plates and counted the appeared colonies. For the NZ9000Erm strain where no nisin was added prior, 4×10^07^ (100%) cells were growing, which decreased to a 0.1% after a 1 hour treatment with 10-fold the IC_50_ concentration of nisin (Figure 3D). The NZ9000NisI strain showed >12–14 times more cells surviving the nisin treatment compared to the NZ9000Erm. But in comparison to the untreated cells 1.4% of the cells were living cells (1 hour incubation with nisin). Interestingly, the number of cells surviving correlates to the incubation time with nisin (Figure 3D). The longer time nisin was present, the fewer the colonies appeared on the agar plate. However, one has to take into account that the nisin concentrations used in this assay varies, since it was adjusted to 10-fold the IC_50_ value. Although the NZ9000NisI strain was treated with 600 nM nisin and the NZ9000Erm strain with just 100 nM, more cells of the NZ9000NisI strain survived.

The number of colonies that appeared when using the NZ9000NisIΔ22 strain was comparable to the number observed with the NZ9000Erm strain, again highlighting the importance of the C-terminus in the immunity mediated by NisI.

This assay showed that a significant number of NZ9000NisI cells were capable of surviving nisin concentrations 10-fold above the IC_50_ value for a certain time period. In clear contrast, the NZ9000Erm and NZ9000NisIΔ22 strains were not able to survive such high nisin concentrations, even when incubated for only 1 hour.

### Phenotype of NisI and NisIΔ22 expressing strains

**Figure 4:**
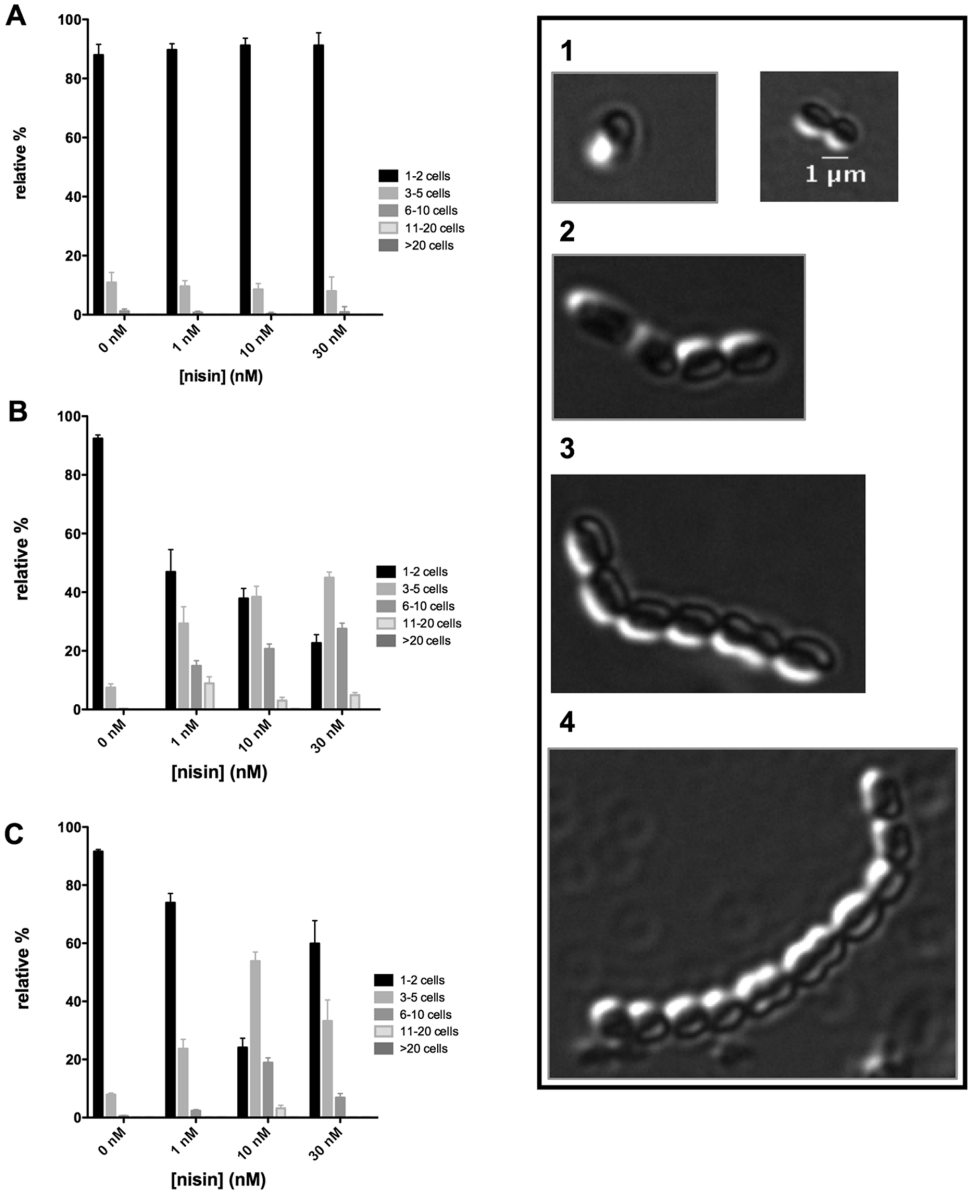
Phenotype visualisation of *L. lactis* cells using the NZ9000Erm, NZ9000NisI and NZ9000NisIΔ22 strain. The different strains were grown until exponential phase (OD_600_ = 0.5). During growth, different concentrations of nisin were added (0, 1, 10 and 30 nM). The cells were transferred and fixed onto a coverslide and the cells were visualised using a Nikon eclipse Ti microscope. The chains were counted and categorized in different classes. Class 1 consisted of 1–2 cocci (black bar), class 2 consisted of 3–5 cells (grey bar), class 3 consisted of 6–10 cells (dark grey bar), class 4 comprised of 11–20 cells (light grey) and class 5 comprised of >20 cells. For each sample the number of counted cells per area was >50. In total, after scanning five different areas at least >500 cell chains were observed.

Different expressing *L. lactis* cells were monitored using increasing concentrations of nisin, *e.g.*, 0, 1, 10 and 30 nM. Here, the growth was measured after 3 hours and the cells were then transferred onto a cover slide and monitored using a Nikon Eclipse Ti microscope. The growth phase of each strain was adjusted to the exponential deviation phase. The chain length was observed and the number of cells forming one chain were counted and grouped according to the number of cells present (Figure 4). In the control experiments (where no nisin was added), the typical double cocci morphology of *L. lactis* was observed for all the strains, NZ9000Erm, NZ9000NisI and NZ9000NisIΔ22 (Figure 4).

For the NZ9000Erm strain, which is highly susceptible to nisin, the majority of chains consisted of double cocci (Figure 4). Even at a nisin concentration of 30 nM, this strain did not change its phenotype.

A similar observation was made for the NZ9000NisI strain, as when no nisin was added, it displayed almost exclusively chains consisting of double cocci (>90% of the population). Upon the addition of 1 nM nisin, the phenotype changed drastically and longer chains were formed in almost 50% of the observed cells in this population. When the nisin concentration was set to 30 nM, a further increase was observed and almost 80% of the cells were localized in long chains. Here, the chain length varied between 3–5 cells (50%) and more than six cells (30%) (Figure 4B). It is worth mentioning here that even longer chains were visible (up to 30 cells in one chain), however a quantitative analysis was not possible as they were mostly lying on top of each other.

For the NZ9000NisIΔ22 strain, in the absence of nisin, more than 90% of the cells were double cocci. The addition of nisin (below the IC_50_) resulted in an increase in the chain length. At a nisin concentration of 10 nM, more than 70% of the cells were localized in chains consisting of 3–10 cells (Figure 4B).

Interestingly, at 30 nM nisin, these longer chains were not observed as frequently. Hence, the majority (>70%) of the cells formed double cocci (Figure 4B) which can be explained by the fact that the addition of nisin at a concentration of 30 nM, is above the IC_50_ value. At this concentration, the NZ9000NisIΔ22 strain is suffering from nisin mediated pore formation as observed by the SYTOx green assay mentioned before.

This data suggests that in the presence of low nisin concentrations in combination with the expression of NisI, resulted in long chain formation of *L. lactis* cells. This phenotype, as observed with the NZ9000NisI as well as the NZ9000NisIΔ22 strains, suggested that this phenotype is mediated by NisI and not *via* its C-terminus.

## Discussion

Several *L. lactis* strains produce the lantibiotic nisin, a 3.4 kDa antimicrobial peptide harbouring five lanthionine rings in its fully active conformation, which are installed by posttranslational modifications [51]. These rings are crucial for the high level of antimicrobial activity as well as its stability [52]. To confer immunity, the nisin producer *L. lactis* strain co-expresses the membrane associated protein systems NisI and NisFEG, which are not localized next to each other on the encoding gene cluster [53] however, both seem to have distinct promoter sites for regulation albeit by the same regulator NisR. Since this regulation is induced by the external addition of nisin, the amount of the immunity proteins in the membrane correlates with the external nisin concentration present in the habitat [32]. The specific NisI promoter is however leaky, thereby ensuring a low but omnipresent immunity [54], which can be up-regulated.

We characterized the function of the homologously expressed nisin specific immunity protein NisI and showed that it can act independently. When over-expressed in *L. lactis*, NisI confers immunity with an IC_50_ value for nisin around 73 nM, which is almost 8–10% of the IC_50_ value determined for the producer strain, where both NisI and NisFEG are expressed (data not shown). The last 22 amino acids are important for the function of NisI, as a deletion of these residues decreased the IC_50_ value to almost one third (22 nM). These results are in-line with the results of Takala *et al.*, which reported a decrease to 22% for the same truncated version [39]. The slight variation could be due to the different experimental setup since they determined MIC values instead of IC_50_. Furthermore, for our study the purification protocol for nisin was performed differently [44].

The ability of nisin to form pores in the membrane of Gram-positive bacteria contributes to significantly to the nM activity of nisin [9]. The fact that NisI seems to inhibit exactly this nisin mediated pore formation as shown with the SYTOx green assay is quite intriguing. This assay has also been used in the past to visualize pore formation, for example, salivaricin 9 from *Streptococcus salivarius*, is also a pore forming lantibiotic [55]. In principle the capturing of nisin by NisI before it reaches the membrane would be sufficient to prevent this pore formation. However, when the nisin concentration raises to a certain point, all NisI proteins in the membrane would be occupied. By further increase in the nisin concentration above this threshold, some nisin molecules could be able to reach the membrane and induce pore formation. However, this is contrary to our observation where NisI can inhibit pore formation even up to 1000 nM nisin (15-fold of the IC_50_) implying that the immunity mediated by NisI is not solely due to the formation of a NisI-nisin complex at the membrane. This consequently raises the question, whether there is an additional function of NisI ensuring the survival of the host cells at high nisin concentrations?

Deletion of the last 22 amino acids of NisI (NZ9000NisIΔ22), leads to pore formation even at lower concentration of nisin indicating that the C-terminus is responsible for the inhibition of the pore formation. The last 22 amino acids have been shown to be sufficient to confer some resistance to nisin when fused to another protein. SpaI-NisI hybrids were created, where the last 22 amino acids of NisI was attached to the SpaI protein which is normally conferring immunity against subtilin in *B. subtilis*. These hybrids were however able to confer resistance to nisin whereas the full-length SpaI protein was not. This shows that only C-terminus of NisI is already enough to confer some resistance against nisin [39]. Although substantially lower, the NZ9000NisIΔ22 strain was still able to confer some resistance. This is also likely arising from a nisin binding event to the rest of the NisI protein. Due to this interaction event, a higher amount of nisin is needed to fulfil the activity of nisin *e.g.* pore formation as reflected by the higher IC_50_ value of the NZ9000NisIΔ22 strain as compared to the NZ9000Erm strain.

Interestingly, in our IC_50_ measurements, no growth was observed at concentrations above 70 nM nisin for the NZ9000NisI strain. Here, we observed the formation of chains consisting of high numbers of *L. lactis* cells (Figure 4). Our data showed that this clustering of cells, which can reach up to 10–20 cells, is directly correlating with the concentration of externally added nisin. An increase in the nisin concentration leads to the formation of longer chains. When NisI was present but no nisin was added only the normal double cocci chains were observed. This showed that when NisI is expressed, the addition of nisin induces a morphological change in the *L. lactis* cells, which is more pronounced at higher nisin concentrations. This is also observed in the NZ9000NisIΔ22 strain suggesting that this is not mediated by the C-terminus, in contrast to the observed pore formation inhibition.

It is worth mentioning that cells with a long chain morphology sediment faster than cells with short chains. This sedimentation can be observed when the NZ9000NisI strain is incubated with high nisin concentrations (data not shown). This long chain formation has been described as the first step towards biofilm formation [56]–[59]. We tested whether the NisI expressing strains indeed form biofilms, however in our hands biofilm formation was not observed (see File S1, Supporting Information).

Interestingly, the long chain formation is reversible, and suggests that the phenotype depends on the external nisin concentration. So when the nisin concentration is to high the expression of NisI induces different phenotype, which ensures the survival of some *L. lactis* cells until the nisin concentration drops again.

One intriguing question still remains: how does the C-terminal part of NisI inhibit pore formation?

In the recently reported SpaI structure, a rather flexible N-terminus has been found which folds upon lipid binding [60]. It is tempting to speculate that such a flexible termini is also present in NisI, where it is localized at the C-terminus. Maybe the C-terminus is binding to or near Lipid II and thereby inhibiting the binding of the nisin molcules to Lipid II, upon which pore formation cannot occur.

A similar inhibition of the nisin-Lipid II binding has been indirectly observed when vancomycin was added prior to nisin. Here, vancomycin was provided first to nisin-sensitive cells. Since Lipid II was occupied with vancomycin, which lacks any pore formation activity, the nisin molecules added afterwards, were not able to bind Lipid II anymore. Subsequently no nisin mediated pore formation was observed [23]. This showed that when Lipid II is occupied by another compound like for example vancomycin, nisin cannot form pores. Similar to this, the C-terminus of NisI might be binding to Lipid II, thereby ensuring that no nisin-Lipid II complex can be formed. Thereby, *L. lactis* becomes immune towards nisin even at concentrations above the determined IC_50_ value. This immunity mechanism of NisI is quite intriguing as it protects *L. lactis* itself from nisin without degrading or damaging it. Not only the inhibition of pore formation is ensured, but also the alternative mechanism of Lipid II displacement by nisin [30] would be circumvented. Moreover, when the concentration of nisin decreases, this NisI - Lipid II interaction appears to be dissociating and at low nisin concentrations, the *L. lactis* cells continue to grow.

## Conclusions

Bacterial strains that produce antimicrobial peptides like lantibiotics must protect their own membrane against the antimicrobial activity of their own peptides. *L. lactis* protects itself against nisin by the expression of two protein systems; NisI and NisFEG. The latter has been shown to expel nisin from the membrane into the extra cellular media [33].

Here, we showed that NisI plays a role in inhibiting nisin mediated pore formation *via* its C-terminus even at very high nisin concentrations. Additionally, the NisI expressing strains form long chain cluster of *L. lactis* cells, which are reversible and counteracts high concentrations of nisin. In the habitat of *L. lactis*, it is likely that such high concentrations of nisin are only present for a short-period of time, since the nisin molecules will diffuse away into the media. During this short time, NisI is however able to confer immunity.

## Supporting Information

File S1 **Supporting Information.**
(DOCX)
