# Supplementary material for: Lantibiotic Immunity: Inhibition of Nisin Mediated Pore Formation by NisI
Source: PLoS One. 2014 Jul 11;9(7):e102246. doi: 10.1371/journal.pone.0102246 (PMC4094520; doi:10.1371/journal.pone.0102246)
Supplement: File S1 — Supporting Information. (DOCX) [file pone.0102246.s001.docx]

**Supplementary information of**

**Lantibiotic immunity: Inhibition of nisin mediated pore formation by NisI**

**Zainab AlKhatib, Marcel Lagedroste, Iris Fey, Diana Kleinschrodt, André Abts and Sander H.J. Smits***

The NZ9000NisI and NZ9000NisI∆22 strains show an long chain phenotype when grown in the presence of nisin (see Figure 4). We examined whether this can be the first step towards biofilm formation. We used a crystal violet-based microtiter plate assay, which has been used for *L. lactis* before to visualize biofilm formation (1). This stain binds to negatively charged molecules, such as peptidoglycan and DNA, and its absorption to cells therefore serves as a measure for biomass at the surface, i.e., biofilms. *L. lactis* was grown for 24 hours in wells of polystyrene (hydrophobic) microtiter plates. Next, the media was removed and the remaining well-associated cells were stained with crystal violet and quantitated. Here, however no difference was observed for the NZ9000erm, NZ9000NisI and NZ9000NisI∆22 strain (Figure S1). When dividing the OD_575_ by the OD_600_ the biofilm formation can be quantitated. This value should be high (> 10) when biofilms were formed. In our experiment this value was always around 1.0, which shows that biofilms are not formed. This suggests that the formation of longer cocci as observed is not subsequently leading to biofilm formation. Rather it seems an effect of the nisin concentration in the media.

Figure S1: After 24 h incubation the biofilm formation ability of different *L. lactis* strains (NZ9000Erm; NZ9000NisI and NZ9000NisI∆22) was tested by a method described by Zaidi *et al.* (1). The resuspended cell pellet left in wells of 96-well microtiter plate shows similar OD_600_ values between the strains and little difference when nisin (50% of the determined IC_50_ value) was added. Staining with crystal violet of the „biofilm“ cells, which remains on the suface of the 96-well plate enables to determine biofilm formation by relating the cell density at 600 nm to the optical density measured at 575 nm. Ratio of OD_575nm_ to OD_600nm_ indicates biofilm formation by high values. For all the tested strains the ratio was around 1.0. Therefore the strains do not form biofilms under the tested conditions.

**Biofilm formation assay**

To test the ability of the different *L. lactis* strains to form a biofilm, depending on the presence of NisI and Nisin was tested using a method described by Zaidi et al. (1). Briefly, an overnight culture of NZ9000Erm, NZ9000NisI or NZ9000NisI∆22 was diluted to OD_600_ of 1 with fresh GM17 media. 200 µl were transferred into a 96-well microtiter plate, supplemented with nisin and incubated for 24 h at 30 °C. Here, the nisin concentration was chosen for every stain to be 50% of the determined IC_50_ value. As a control the same experiment was performed with no nisin was added. This was done in duplicates.

After 24 hours at 30 °C the GM17 medium was carefully removed and for one row on the 96-well plate, the cells, which stick onto the well were resuspended with fresh GM17 medium. The OD_600_ was measured and are termed „biofilm“ cells. The second row for every strain was washed 3 times with dionized water and dried for 24 hours. The cells were stained for 15 min with 0.1 % crystal violet solution (Sigma Aldrich), rinsed with water and dried overnight. The stained cells were dissolved in 200 µl DMSO and the OD_575_ was measured. The ratio of “biofilm” cells to stained cells was calculated and should in principle indicate biofilm formation. Steril GM17 served as a control and background. This assay was performed several times with independently grown cultures in at least 6 replicates.

**References:**

1. Zaidi, A. H., Bakkes, P. J., Krom, B. P., Mei, H. C. Van Der, & Driessen, A. J. M. (2011). Cholate-Stimulated Biofilm Formation by *Lactococcus lactis* Cells. Applied and Environmental Microbiology, 77(8), 2602–2610. doi:10.1128/AEM.01709-10

2. Burmolle, M., et al. 2006. Enhanced biofilm formation and increased resistance to antimicrobial agents and bacterial invasion are caused by synergistic interactions in multispecies biofilms. Appl. Environ. Microbiol. 72:3916– 3923
